# Digital Tools as Parental Support—A Study Protocol Describing Prospective Development and Exploration of Two Digital Tools for Parents

**DOI:** 10.3389/fdgth.2021.698969

**Published:** 2021-11-24

**Authors:** Caroline Bäckström, Henrik Engström, Rajna Knez, Margaretha Larsson

**Affiliations:** ^1^School of Health Sciences, University of Skövde, Skövde, Sweden; ^2^School of Informatics, University of Skövde, Skövde, Sweden; ^3^Skaraborg Hospital, Skövde, Sweden

**Keywords:** digitalization, professional support, pregnancy, childbirth, labor, parenting

## Abstract

**Background:** The access to digital tools for parents is increasing, and further exploration is needed to gain knowledge about parents' experiences in using such tools, for example, when preparing for childbirth and parenthood. This study protocol describes a prospective study that will explore serious games as digital tools for parental support, and both parents' and healthcare professionals' views will be included. The objectives of the prospective study are to explore two different serious games: (1) Childbirth Journey (Swedish: Förlossningsresan), relating to pregnancy, childbirth and parenthood; and (2) Interplay (Swedish: Samspel), relating to parental couple relationships and parenthood.

**Methods:** An intervention study will be conducted. The study will include four different sub-studies (A–D) with both qualitative and quantitative methods and a longitudinal design. Both parents (A, B and D) and healthcare professionals (C) will be included, and data will be collected through interviews (A–C) and repeated web-based questionnaires (D). Data will be analysed using phenomenography and qualitative content analysis (A–C), and descriptive and analytical analyses will be performed for comparisons and associations (D).

**Discussion:** The value of monitoring and reporting on developments and trends in digital innovation for public health has been stipulated by the World Health Organization. The prospective study will contribute further knowledge about multidisciplinary development of digital tools as professional support for parents, as well as knowledge about parents' and healthcare professionals' experiences using digital tools concerning pregnancy, labour, parenthood and parental couple relationships.

**Trial Registration:** This study was retrospectively registered (02/10/2020) within the ISRCTN with ID: ISRCTN18017741. http://www.isrctn.com/ISRCTN18017741.

## Background

Expectant and new parents turn to digital sources, such as the Internet, to obtain information about pregnancy, labor, and parenthood (1–3), but they sometimes find it difficult to evaluate the trustworthiness of the information (4–6). Furthermore, the development of digital technology in society is rapid (7), and digital tools available for parents are, sometimes, unexplored. For example, in Denmark, a serious game was developed by Region Midt/Hospitalsenheden West, Denmark (Danish title: *Samspil*), with the intention of strengthening new parents' couple relationships and cooperation within parenthood. Since 2019, *Samspil* has been offered to new parents in Denmark, and an evaluation has shown that both parents and professionals express interest in using it (8). However, there is a lack of research on parents' and professionals' experiences using *Samspil* or other serious games as digital tools regarding childbearing. This study protocol describes a prospective study that will provide additional perspectives on how parents' couple relationships and parenting can be supported with digital tools.

Expectant parents experience one of the most drastic life changes for the human being—the transition to parenthood, and they pose, usually, issues on both medical and practical matters (9). In recent years, it has become increasingly common for expectant parents to turn to digital sources for health information (1, 2). Overall, there has been rapid development of advanced technology in society, and research shows that humans are experiencing that the rapid integration of digital technology affects both their personal and professional lives (7). Nevertheless, expectant parents search for health-related information online, and the Internet plays an important role in providing them with support (3). One literature review revealed that most expectant mothers have access to the Internet, and they use it to retrieve information about pregnancy, childbirth, and the expected child (10). In addition, expectant fathers have been shown to obtain Internet-delivered information related to parenting, how to support their pregnant partner, and how to promote theirs and their family's well-being and health (11). Both expectant mothers and fathers also use the Internet to read about others' experiences of giving birth to a child and becoming a parent (12, 13). Previous research shows that parents want information on the Internet to be immediate, regular, detailed, and entertaining. Also, they prefer information provided by health care professionals and information that is practical and reassures their knowledge (1). In a recent study, Bäckström et al. (14) showed that parents perceive that future digital parental support should provide them with opportunities to have both virtual and in-person meetings, as well as individualized support based on professional knowledge and trustworthiness. The authors claim that such future digital parental support might facilitate digital health literacy of new and would-be parents. However, further exploration is needed to gain knowledge of how digital tools should be designed so that parents could benefit from using them.

The way expectant parents seek information has been described as a “holistic learning process to seek meaning”, and it affects their decision-making process regarding pregnancy (15). For example, expectant mothers' decision-making process has been shown to involve seeking, collecting, and assessing information from healthcare professionals, other expectant mothers, and research. They base their decisions regarding pregnancy on their perceptions of safety and trustworthiness regarding the information obtained (6). Nevertheless, parents' access to digital tools is increasing (1–3, 7), and further exploration is needed to gain knowledge, for example, about parents' experiences using such tools when preparing for childbirth and parenthood.

Expectant parents are usually cared for within antenatal care. Internationally, which healthcare professionals are responsible for antenatal care varies. In Sweden and several other countries, midwives are the primary caregivers (16). However, it has been revealed that expectant parents tend to use the Internet to verify information provided by midwives within antenatal care (4, 5). Therefore, it is arguable that, instead of disregarding the use of the Internet as a source of information during pregnancy, healthcare professionals, such as midwives, should keep up to date and give expectant parents links to high-quality sites (3). The limited time for antenatal appointments with healthcare professionals can influence expectant mothers' Internet use (15), and there is a difference in how Swedish midwives refer expectant parents to information *via* the Internet (17). A reason why midwives do not refer parents to the Internet could be a lack of Internet-based sources that are deemed trustworthy or interesting to use (18). However, another reason could be a lack of Internet-based sources that are deemed trustworthy or interesting to use. Therefore, further research about digital tools for expectant and new parents is needed to gain knowledge about the content and design of digital tools to be able to promote them.

### Serious Games

Games have been a part of human culture since the dawn of civilization. The Dutch historian Huizinga even argued that games have played a central role in the development of societal structures, such as our legal system (19). Games have been used for education and training for a long time: Chess and Go have been used to teach military strategy (20), and roleplaying has been used in vocational training in many areas, including nursing education (21). The use of digital games for training started almost at the same time as the first digital games appeared in the second half of the twentieth century. Different terms have been used to describe the use of games for purposes other than pure amusement: *edugames, edutainment, learning games, games for change, educational games*, and *serious games*. In the last decade, the term *gamification* has become popular (22). The underlying principle for all of these approaches is that the motivational and recreational elements in games should be leveraged to achieve some additional goal. To a large extent, research relating to games has focussed on studies of serious games (23). In this study, we use the term *serious game* as an umbrella that covers the other approaches (i.e., edugames, edutainment, learning games, games for change, and educational games).

Two aspects that are commonly overlooked in serious games research are how to create an interesting *gaming experience* and how to *evaluate the experience* users have when playing (24). One characteristic found in most games providing gameful experiences is that they offer players challenges to overcome. Different players will have different skills and experiences, which means that it is impossible to create a game that fits all players (24). If a game is too hard, players will give up; if it is too easy, they may lose interest (25).

The focus in research studies is mainly on evaluating the effect of the game in terms of the serious goal; for example, to determine its learning or rehabilitation effect. However, Engström (26) and Martin (23) argued that there is a gap between research and the game industry. The process of game development, as it is conducted in the game industry, is not well understood. The deep interdisciplinary collaboration at game companies is rarely reflected in academia, which is organized in disciplinary structures. It becomes problematic when games are developed by researchers who lack experience in conventional game development but still approach the more difficult challenge of creating a serious game. Such approaches risk producing a game that has a good representation of the serious element but fails to give a satisfying player experience—hence, the original motivation for using games is lost. On the other end of the spectrum, there are examples of serious games that manage to give players an enjoyable experience to the extent that they forget about the context in which the game is being played. If a learner enters *gamer mode* (27), this may compromise the pedagogical goals of an exercise. An important conclusion that can be drawn from the multitude of previous studies of serious games is that they are challenging to create (28). Recent research has shown that expectant and new parents ask for more digital tools, such as serious games (14), and there is a lack of knowledge regarding serious games for expectant and new parents. Therefore, the prospective study described in this study protocol will explore serious games as digital tools for parental support, and both parents' and healthcare professionals' views will be included.

This study protocol describes a prospective study in which we will strive to tackle the challenges identified above. First, we will do this by acknowledging that they exist, which is not always done in serious games projects. Second, the research team will be composed of a mix of researchers with different backgrounds and participants with experience of game development. Within the study, two different serious games will be explored. One of them, *Childbirth Journey*, is newly developed, and no exploration of the game has previously been conducted. The other, *Interplay*, will be further developed from the original Danish version and reworked to correspond to the Swedish context. The two different serious games represent different characteristics: *Childbirth Journey* is a story-driven game focussed on conveying information facilitating expecting parents' preparation for childbirth and parenthood, while *Interplay* is a casual quiz game mostly focussed on one central message—that it is valuable to nurture one's couple relationship. Further, *Interplay* includes information about couple relationship and parenthood (this will be further described in the methods section). Subsequently, by exploring two serious games representing different characteristics and different stages of development, we will see diverse views, and thus, gain a deeper understanding about how digital tools should be provided to expectant and new parents. Both serious games will be developed in collaboration with game companies.

## Objectives

The overall aim of the prospective study is to explore two different serious games, which are as follows: (1) *Childbirth Journey* (Swedish title: *Förlossningsresan*), relating to pregnancy, childbirth and parenthood; and (2) *Interplay* (Swedish title: *Samspel*), relating to parental couple relationships and parenthood. The secondary aims of the prospective study are to explore the following:

(1) Parents' perceptions of and user behavior in *Childbirth Journey*. Research question: How do parents perceive *Childbirth Journey* as a digital parental support relating to pregnancy, childbirth and parenthood? (Study A).
(2) Parents' perceptions of and user behavior in *Interplay*. Research question: How do parents perceive *Interplay* as a digital parental support relating to parental couple relationships and parenthood? (Study B).
(3) Healthcare professionals' perceptions of *Childbirth Journey* and *Interplay*. Research questions: (1) How do healthcare professionals perceive *Childbirth Journey* as a digital support for parents relating to pregnancy, childbirth and parenthood, and (2) How do healthcare professionals perceive *Interplay* as a digital support for parents relating to parental couple relationships and parenthood? (Study C).
(4) Parents' user behavior over time and individual sessions in *Interplay*, as well as their perceived quality of the parental couple relationship, social support, and sense of coherence related to the results in study A–C (study D).

## Methods and Design

This study protocol describes a prospective study that is explorative, and mixed methods with both inductive and deductive approaches will be used. A mixed method study, according to Creswell [(29), p. 457], “is one in which the research uses at least one quantitative method and one qualitative method to collect, analyze, and report findings in a single study”. Before designing the prospective study, a systematic literature review (submitted for review) and interviews with expecting and new parents were carried out (14) to broaden our understanding about how expecting and new parents use digital sources regarding childbearing and parenthood. The prospective study is an intervention study that includes the exploration of two different serious games, *Childbirth Journey* and *Interplay*. This prospective intervention study will include four different sub-studies (A–D), using both qualitative and quantitative methods and a longitudinal design. The qualitative studies (A–C) will be located in a county in southwest Sweden. The quantitative study (D) will be located nationally in Sweden. Participants to be included are as follows: expectant parents (A and B), new parents (D), and healthcare professionals at antenatal clinics and within family counseling (C). The different designs to be used within studies A–D are presented in Table 1. This study uses a QUAL-QUAN *sequential mixed design* according to the classification presented by Schoonenboom and Johnson (30), and it was retrospectively registered (02/10/2020) within the ISRCTN with ID: ISRCTN18017741. http://www.isrctn.com/ISRCTN18017741.

**Table 1:** Overview of studies including methods, interventions, sample, data collection and data analysis.

**Study**	**Methods**	**Intervention**	**Sample**	**Data collection**	**Data analysis**
A	Qualitative method	Childbirth journey	10 parents	Interviews	Phenomenographic analysis
B	Qualitative method	Interplay	10 parents	Interviews	Phenomenographic analysis
C	Qualitative method	Childbirth journey and interplay	10-healthcare professionals	Interviews	Phenomenographic analysis
D	Quantitative method, prospective longitudinal	Interplay	120 parents	Repeated questionnaires	Descriptive and analytical analysis

### Interventions

For the interventions, two different serious games, *Childbirth Journey* and *Interplay*, are to be explored. All participants will receive one of the two serious games, depending on which part of the study they participate in. Participants in studies A and C will receive *Childbirth Journey*, and participants in studies B, C, and D will receive *Interplay*. The two different serious games are described in greater detail below.

#### Childbirth Journey

One of the serious games to be explored in this prospective study, *Childbirth Journey*, is newly developed, and no exploration of the game has previously been conducted. *Childbirth Journey* is a story-driven game. This means that the main reward from such gameplay is to experience the resulting narrative. Storytelling in games, however, poses unique challenges as it contrasts with traditional drama:

*It is a difficult balancing act between what is perhaps the biggest challenge in relation to storytelling in games: the conflict between the strong player control required for a good interactive experience and the strong author control traditionally required for good drama* (31).

The idea of serious games design is that expectant parents will be able to choose different types of scenarios for the characters to experience in the game. When expectant parents are controlling the different scenarios, they can process any concerns or fears according to their individual needs. Based on previous research on expectant parents' needs of information (1–3, 10–13), the different scenarios included deal with antenatal care; expectant parents' preparation at home; the start of labor; labor; breathing techniques and pain relief; breastfeeding and formula; postnatal care; and the postpartum period, including the first days at home with the child. *Childbirth Journey* does not pose any real obstacles to player progression. The goal of developing *Childbirth Journey* was to create a digital tool as parental support for expectant parents that could facilitate expectant parents' knowledge about preparing for childbirth and parenthood hence professional support previously has been shown to facilitate expectant parents' knowledge and understanding (32).

The content in *Childbirth Journey* is developed and controlled by healthcare professionals in antenatal, labor, postnatal, and child health care, as well as a researcher specializing in serious games, creating a multidisciplinary team. Since *Childbirth Journey* is provided digitally, this enables expectant parents in Sweden to use it without hindrance, regardless of time and space. The basis for developing *Childbirth Journey* was previous research conducted by the researchers (32), as well as interviews with expectant and new parents about their use and experiences of digital tools in relation to pregnancy, labor, and parenthood. The results of the interviews is published elsewhere (14). *Childbirth Journey* will consist of one interface for the pregnant woman and another for the partner because previous research has shown the importance of including both parental roles in professional support (32). *Childbirth Journey* will be provided both as a mobile application and as a computer version, and there will be Swedish and English versions.

In study A, expectant parents will take part in the intervention, and their experiences from and user behavior in *Childbirth Journey* will be explored. In study C, healthcare professionals will take part in the intervention, and their experiences of *Childbirth Journey* will be explored.

#### Interplay

The second serious game that will be explored for this study, *Interplay*, represents another game genre, the casual quiz game (33), where competition is a catalyst for social interaction. Many similar games contain puzzles that the player must solve to progress. These can potentially provide the player with an interesting challenge, but they may also stop a player from continuing. However, *Interplay* does not pose any real obstacles to player progression, as by focussing on the core aesthetics of the genre, we reduce the risk that some players might become stuck. *Interplay* has been used by many players in its Danish version (Danish title: *Samspil*), so the design is well tested, although it has not been explored in any research study.

In preparation for this study, the Danish version has been translated to Swedish and revised to be suitable within the Swedish context. *Interplay* includes concrete tasks and learning elements that aim to strengthen expectant and new parents' knowledge and ability to handle their role as partners and parents. The intention is that parents should use *Interplay* to help in creating a safe and well-functioning environment for them and their children. Since *Interplay* is provided digitally, this enables parents to use it without hindrance, regardless of time and space.

In study B, expectant parents and new parents will take part in the intervention, and their experiences from and user behavior in *Interplay* will be explored. In study C, healthcare professionals will take part in the intervention, and their experiences of *Interplay* will be explored. The results of study B and C will contribute with further development of the *Interplay* before conducting study D. In study D, expectant and new parents will take part in the intervention, and their user behavior over time and individual sessions in *Interplay*—as well as associated factors between parents' user behavior and experiences from *Interplay* and their perceived quality of parental couple relationships, social support, and sense of coherence—will be explored.

### Participants

In both studies A and B, parents will be recruited through midwives at antenatal units and with flyers and ads on social media. We aim to include ~10 parents in each case in studies A and B; the targeted participants for each study are described in Table 1. In study C, ~10 healthcare professionals (i.e., midwives in antenatal care, nurses in child health care, and family supporters in family counseling) will be recruited through email. The number of targeted participants in studies A–C is considered appropriate to gain trustworthy knowledge about the phenomena explored within the contextual circumstances for the research participants, hence qualitative research does not intend to generalize the results into other settings (34). Based on power calculations, ~120 parents will be included in study D. Quantitative calculation for power strength is based on an expected relationship of 0.4, which requires at least 100 participants, given power of 80% and significance level 0.05 (35). In study D, a total of 120 participant is sought to ensure power. Participants will be recruited through flyers and ads on social media. In the studies (A–D), both convenience and snowball sampling will be used. The *inclusion criteria* are as follows: (1) expectant and new parents in the study setting (A and B); (2) healthcare professionals at antenatal units and in family counseling in the study setting (C); and (3) expectant and new parents in Sweden (D). Inclusion in the study is regardless of whether the pregnancy or birth is high or low risk, or if the baby is born premature or with illness, which means that parents are included on equal terms as expecting or new parents. The *exclusion criteria* are as follows: (1) parents with insufficient ability in speaking, reading, or understanding Swedish (B and D) or English (A); (2) parents or healthcare professionals who do not have access to mobile phones that enable digital parental support to be used [B, C (*Interplay* intervention), and D]; (3) parents or professionals with severe visual impairment or other physical disorder that would prevent them from using the touch feature on mobile phones [B, C (*Interplay* intervention), and D]; and (4) single parents or parental couples in which only one of the parents agrees to participate (B and D).

### Data Collection

For this study, we will collect data through interviews (A–C) and repeated web-based questionnaires (D). In studies A and B, interviews will be conducted with parents individually or with two parents within a couple relationship together, depending on the preference of the parents. In cases when interviews are held with the parental couple together, their listening to each other may make them aware of things they might not otherwise have reflected on, which might contribute to broader narratives. In study C, focus-group interviews with healthcare professionals will be conducted. Interviews will be held when the participants have had access to the intervention (*Childbirth Journey* or *Interplay*) for ~2 weeks. Interviews will follow interview guides with questions regarding user experience and usage of the serious games according to such themes as trustworthiness, usefulness, support, knowledge, and feelings related to labor, parenthood, and parental couple relationships. Examples of questions are as follows: “*Please describe your experiences from using* Childbirth Journey”, “*Did you perceive it as easy to understand?*”, “*Has* Childbirth Journey *affected you somehow in your preparation for labor?*” (study A); “*Has* Interplay *affected your couple relationship somehow?*”, “*Did you experience* Interplay *as a support in your couple relationship or parental role?*” (study B) and “*Will you offer* Childbirth Journey *(or* Interplay*) for expectant parents?*” (study C). Follow-up questions will be used, such as “*Could you please tell me more about that?*” and “*Please describe this further*”. Besides questions regarding the participants' previous experience from using serious games will be included in the interview guides. The interview guides have been pilot tested with results showing that the interview questions were easy to understand and respond to, by the interviewed. The pilot interviews will not be included in the data analysis in each study (A–C). The interviews will be held by researchers with doctoral degrees and previous experience in qualitative methods, as well as individual and focus-group interviews. The interviews will be audio-recorded and transcribed verbatim.

In study D, participants will complete a web-based questionnaire at baseline (Q1) before receiving the intervention. Another web-based questionnaire (Q2) will be sent to the participants 6 months after Q1. Two reminders will be sent to participants who do not complete Q1 or Q2; the reminders will be sent at 1-week intervals. Since study D will be based on the results of studies B and C, the measures and questions to be included in the questionnaires will be decided after publication of this study protocol; however, questions regarding participant socio-demographics will be included in Q1, which is important since this is a national survey. In addition, some previously validated measures will be included in both Q1 and Q2, as described below. Since *Interplay* includes concrete tasks and learning elements aiming to strengthen expectant and new parents in their knowledge and ability to handle their role as partners, we will include the QDR36 questionnaire (36) in Q1 and Q2. The QDR36 consists of 36 items covering five different dimensions of the parent dyad: *consensus, cohesion, satisfaction, sensuality*, and *sexuality*. In addition, parents' perceived quality of the couple relationship has previously been shown to be associated with their sense of coherence [SOC; (37)]; therefore, the validated Swedish version (38) of the SOC-13 (39) will be included in Q1 and Q2. SOC-13 consists of 13 items covering three different dimensions, comprising parents' senses of *comprehensibility, manageability*, and *meaningfulness*. Furthermore, parents' perceived quality of the couple relationship has previously been shown to be associated with their perceived social support (37). Therefore, the Swedish version (40) of the multidimensional scale of perceived social support [MSPPS; (41)] will be included in the questionnaires (Q1–Q2) to assess the parents' perceived social support. The scale consists of 12 items that cover three different dimensions of social support—support from *family, friends*, and *significant others*. The measures included in the questionnaires for this study are published elsewhere.

For studies B–D, log data for process evaluation purposes during the intervention will be collected. These data will be utilized to evaluate the usage of the mobile application; for example, through login and logout times, the activities completed, and the time spent studying each content area or answering questions related to it. Only pre-specified log data will be collected, and they will not contain any information that can be used to identify individual participants. This means that IP addresses will not be recorded. Participants' mobile application usage will only be studied and presented through aggregated metrics, such as average session duration.

### Data Analysis

#### Qualitative Data Analyses

The qualitative data from interviews with parents and professionals will be transcribed verbatim and analyzed using phenomenography (42). When analyzing the interviews (A–C), phenomenographic analysis will be conducted to explore the participants' various ways of understanding their experiences from taking part of the interventions *(Childbirth Journey* and *Interplay)*. Within phenomenography, peoples' various ways of perceiving or understanding a phenomenon are explored, not the actual phenomenon itself, as in many other qualitative methods (42). For study A–C, data analysis will be carried out using the seven steps described by Sjöström and Dahlberg (42): familarisation, compilation, condensation, grouping, comparison, naming and contrastive comparison. Data will be scrutinized within the research team, which consists of researchers with complementary experiences from qualitative research on professional support and serious games, who will be able to judge the data interpretation. The results will be published in international scientific journals.

#### Statistical Analyses

Participant answers to the questionnaires will be transferred directly into the Statistical Package for the Social Sciences (SPSS), in which both descriptive and analytical analyses will be performed for comparisons and associations. Analyses will be controlled for such covariates as gender, age, education, marital status, length of parental couple relationship, and perceived economy. An alpha level of ≤ 0.05 will be accepted as significant. The prospective sample size is deemed to provide adequate statistical power for the analyses to be performed.

## Discussion

This study protocol describes a prospective development and exploration of two digital tools. Both *Childbirth Journey* and *Interplay* are digital tools that we are planning to introduce to parents and healthcare professionals in Sweden. They are quite unique and innovative among the digital parental tools available related to pregnancy, childbirth, and parenthood. *Childbirth Journey* is specific because it is designed as a story-driven educational game with medical content that is reviewed by medical experts in the field, where the main story follows the journey from early pregnancy to early parenthood. *Interplay*, in contrast, represents a casual quiz game that catalyses partner interaction. The intention of *Childbirth Journey* is to mirror parents' questions and provide answers, information, and resources in order to directly educate them. In addition, *Childbirth Journey* may indirectly help parents develop a sense of control over an unknown life situation, and consequently, even foster a feeling of higher serenity. In this way, *Childbirth Journey* is not the journey itself, but rather, it serves as guidance and a companion for parents who travel down this road in real life.

Involving interdisciplinary professionals and consumers in early product design is critical in order to ensure that the final product is usable and acceptable, as well as that it will lead toward healthy behaviors (43). *Childbirth Journey* represents just that: a digital tool developed by multidisciplinary medical professionals with initial input from expectant mothers and partners. Furthermore, healthcare professionals should support the evaluation of existing parenting applications to ensure that parents are acquainted with the most effective and up-to-date options (44). Obtaining healthcare professional experiences of the digital tool in our study will ensure that *Childbirth Journey* is presented to professionals, but most importantly, their feedback on the tool will bring valuable material that, together with the parents' experiences, will be evaluated and utilized in finalizing the product. Beyond the experience obtained with *Childbirth Journey*, parents will have the possibility to use the other digital tool in our study, *Interplay*, which has the intention to enhance new parents' couple relationships and cooperation within parenthood. As mentioned, the two serious games that are to be explored for this study represent different characteristics; one is a story-driven educational serious game (*Childbirth Journey*) focussed on conveying information controlled by healthcare professionals, the other is a casual quiz focussed on interaction and communication between parental couples (*Interplay*). Because the two serious games represent both different characteristics and different stages of development, we will be able to access diverse views and gain a deeper understanding of how digital tools should be provided to expectant and new parents.

Parenting can be challenging (44), and the transition to this period can be stressful for new parents (45), these aspects can be potential limitations as they can hinder parents from participation in the studies described in this study protocol. Identifying factors that may have an impact on parental adjustment is necessary to provide an intervention programme aimed at alleviating this period of transition by targeting those aspects specifically. For some parents, parental couple relationship quality may decrease during pregnancy (37), and the results of a recently published study suggest that prenatal couple interaction quality may predict the parents' perceptions of their spouses' parenting at 8 months after childbirth (46). The co-parenting relationship, defined as the way in which parents work together in their roles as parents, has been associated with parenting and parental adjustment, as well as child outcomes (47). Moreover, even long-term implications of co-parenting quality for adult and child outcomes have been suggested (48). A randomized controlled trial in which expectant parents received professional support by midwives proved that professional support can strengthen parents' perceived quality of their couple relationships 6 months after birth (49). In addition, expecting parental couples who attend professional support together (such as parental groups with other expecting parents and midwives) are less likely to commit couple separation the first 2 years after birth (50). Consequently, we might say that during the transition period and in early parenthood, it is desirable to provide care interventions that will focus on the quality of couple interactions and co-parenting aspects of the parent's journey, as well as on the parents' perceptions of each other's parenting. *Interplay* is a digital tool that accounts for these aspects and includes concrete tasks and learning elements aiming to facilitate parents' interactions and strengthen their ability to handle their roles as both parents and partners. *Interplay* may be seen as a digital tool providing a virtual space that invites partners to weave together an unusually fine fabric that will embrace their children; a matrix or a nest in which the newborn can land softly, warmly, and safely. This portrayal can serve as a metaphor for interpersonal space built on solid ground made up of the perceived quality of the parental couple relationship and their social support, as well as their SOC. However, parents' experiences, as well as parents' user behavior from using *Interplay*, should be explored to prove its benefits.

The value of monitoring and reporting on developments and trends in digital innovation for public health has been stipulated by the World Health Organization (51). This study will contribute further knowledge pertaining to the multidisciplinary development of digital tools as professional support for parents, as well as knowledge regarding parents' and healthcare professionals' experiences using digital tools concerning pregnancy, labor, parenthood, and parental couple relationships. In addition, the results of this study might work as an indicator or a guide for healthcare professionals in their work with understanding how to meet the behavior and demands among parents (15). In their study, Bäckström et al. (14) claims that healthcare professionals should have an active role in developing digital parental support. Therefore, the design of the previous study C including healthcare professionals who receive the interventions, could be seen as a study strength. The knowledge obtained from the studies included (A–D) will be used to further develop both *Childbirth Journey* and *Interplay*. Thereafter, we plan to conduct a randomized controlled trial in which parents' effects from using the additional developed *Childbirth Journey* and *Interplay* will be evaluated. One possible limitation to this study could derive from a lack of interest from parents and professionals to participate since participation involves taking part and using an intervention, which might be considered as time-consuming. Besides, both *Childbirth Journey* and *Interplay* should be seen as prototypes for two different serious games not previously offered to expectant or new parents nor professionals, hence currently we lack knowledge on how parents and professionals may experience them. Besides, *Childbirth Journey* will only be sparsely tested by parents and healthcare professionals before conducting studies A and C. Therefore, knowledge about user experience and problematic experiences with technology will be limited when recruiting participants for the studies. For studies B, C and D, such limitations are not as tangible since the original Danish version of *Interplay* (i.e., Samspil) has been used by parents for a longer time period, which could be seen as a study strength. Therefore, the prior participant information will include time-aspects and information clarifying that the interventions should be seen as prototypes. The prior informant information will be provided both in written and verbally. Strengths, on the other hand, are that the prospective studies include mixed methods which will provide broad knowledge about the interventions, and the research group consist of a multi-disciplinary team with experiences of mixed methods and intervention studies.

## Ethics and Dissemination

Interventions included in this study are considered low-risk interventions. A limited number of expected negative experiences for the participants may become relevant. These may relate to usage of the serious games, problematic experiences with technology, negative feelings regarding a participant's couple relationship with their partner (studies B and D), or insufficient support for the upcoming labor and parenthood. In contrast, the use of the serious games may lead to positive effects on participants' feelings of being able to handle childbirth, parenthood, or the couple relationships with partners (studies A, B, and D). For example, expectant parents may perceive the interventions (*Childbirth Journey* and *Interplay*) as digital tools, and deem the information as trustworthy, because they are controlled and provided by healthcare professionals. Negative side effects, such as disappointment, and participant dropouts are to be examined, and we will use both qualitative and quantitative research to detect any possible deterioration, as well as in the subjective experiences of the participants. The prospective study shall follow the ethical regulations and guidelines outlined in Swedish law and the Code of Ethics of the Declaration of Helsinki (52). Personal data shall be processed in accordance with the EU General Data Protection Regulation (53) and only the research manager has access to the participant identities. The participation is voluntary and written informed consent shall be obtained from all participants. The participant identities will be handled with confidentiality and the data material will be stored in ways that only allow the researchers access. The participants will not be provided any reimbursement for participation in neither of the studies. This study has been approved by the Regional Ethical Review Board in Gothenburg, Sweden (Dnr: 2020-01689).

## Ethics Statement

The studies involving human participants were reviewed and approved by the Regional Ethical Review Board in Gothenburg, Sweden (Dnr: 2020-01689). The patients/participants provided their written informed consent to participate in this study.

## Author Contributions

All authors listed have made a substantial, direct, and intellectual contribution to the work and approved it for publication.

## Funding

This study has been funded by the University of Skövde, Sweden and Skaraborg Hospital, Skövde, Sweden.

## Conflict of Interest

The authors declare that the research was conducted in the absence of any commercial or financial relationships that could be construed as a potential conflict of interest.

## Publisher's Note

All claims expressed in this article are solely those of the authors and do not necessarily represent those of their affiliated organizations, or those of the publisher, the editors and the reviewers. Any product that may be evaluated in this article, or claim that may be made by its manufacturer, is not guaranteed or endorsed by the publisher.

## Abbreviations

MSPPS: Multidimensional scale of perceived social support
SOC: Sense of coherence
QDR36: Quality of dyadic relationship
Q1: Questionnaire at baseline
Q2: Web-based questionnaire.
